# Clinical outcome of children with fluid-refractory septic shock treated with dopamine or epinephrine. A retrospective study at a pediatric emergency department in Argentina

**DOI:** 10.5935/0103-507X.20200092

**Published:** 2020

**Authors:** Guillermo Kohn-Loncarica, Ana Fustiñana, César Santos, Guadalupe Paniagua Lantelli, Hernan Rowensztein, Sebastián González-Dambrauskas

**Affiliations:** 1 Unidad Emergencias, Hospital de Pediatria “Prof. Dr. Juan P. Garrahan” - Buenos Aires, Argentina.; 2 Dirección Asociada de Docencia e Investigación, Hospital de Pediatria “Prof. Dr. Juan P. Garrahan” - Buenos Aires, Argentina.; 3 Red Colaborativa Pediátrica de Latinoamérica (LARed Network) - Montevideo, Uruguay.; 4 Cuidados Intensivos Pediátricos Especializados, Casa de Galicia - Montevideo, Uruguay.

**Keywords:** Sepsis, Shock, septic, Dopamine, Epinephrine, Cardiovascular agents, Child, Sepsis, Choque séptico, Dopamina, Adrenalina, Fármacos cardiovasculares, Niño

## Abstract

**Objective:**

To analyze the clinical outcome of children with fluid-refractory septic shock initially treated with dopamine or epinephrine.

**Methods:**

A retrospective cohort study was conducted at a pediatric emergency department of a tertiary hospital. Population: children admitted because of fluid-refractory septic shock. Clinical outcome was compared between two groups: Dopamine and Epinephrine. Variables evaluated were use of invasive mechanical ventilation, days of inotropic therapy, length of hospital stay, intensive care stay, and mortality. For numerical and categorical variables, we used measures of central tendency. They were compared by the Mann-Whitney U-test and the (2 test.

**Results:**

We included 118 patients. A total of 58.5% received dopamine and 41.5% received epinephrine. The rate of invasive mechanical ventilation was 38.8% for epinephrine versus 40.6% for dopamine (p = 0.84), with a median of 4 days for the Epinephrine Group and 5.5 for the Dopamine Group (p = 0.104). Median time of inotropic therapy was 2 days for both groups (p = 0.714). Median hospital stay was 11 and 13 days for the Epinephrine and Dopamine groups, respectively (p = 0.554), and median stay in intensive care was 4 days (0 - 81 days) in both groups (p = 0.748). Mortality was 5% for the Epinephrine Group versus 9% for the Dopamine Group (p = 0.64).

**Conclusions:**

At our center, no differences in use of invasive mechanical ventilation, time of inotropic therapy, length of hospital stay, length of intensive care unit stay, or mortality were observed in children admitted to the pediatric emergency department with a diagnosis of fluid-refractory septic shock initially treated with dopamine versus epinephrine.

## Abstract (es)

**Objetivo:**

Analizar la evolución clínica de niños con shock séptico refractario a volumen tratados inicialmente con dopamina o adrenalina.

**Métodos:**

Estudio de cohorte retrospectivo con ámbito en un servicio de urgencias pediátrico de un hospital de tercer nivel. Población: niños ingresados con shock séptico refractario a volumen. Se comparó la evolución clínica entre 2 grupos: Grupo Dopamina y Grupo Adrenalina. Las variables de interés fueron: uso de ventilación mecánica invasiva, días de inotrópicos, estancia hospitalaria, estancia en cuidados intensivos y mortalidad. Para variables numéricas y categóricas utilizamos medidas de tendencia central y para comparación las pruebas de U Mann Whitney y χ2 test.

**Resultados:**

Incluimos 118 pacientes. El 58,5% recibió dopamina y 41,5% adrenalina. El uso de ventilación mecánica invasiva fue 38,8% para adrenalina versus 40,6% para dopamina (p = 0,84) con una mediana de 4 días para adrenalina y 5,5 para dopamina (p = 0,104). La mediana para días de inotrópicos fue de 2 días para ambos grupos (p = 0,714). La mediana de estancia hospitalaria fue de 11 para adrenalina y 13 para dopamina (p = 0,554) y de estancia en cuidados intensivos se registró una mediana de 4 días (0 - 81 días) siendo igual en ambos grupos (p = 0,748). La mortalidad fue de 5% para el Grupo Adrenalina versus 9% para el Grupo Dopamina (p = 0,64).

**Conclusiones:**

En nuestro centro, no observamos diferencias en uso de ventilación mecánica invasiva y tiempo de inotrópicos, estancia hospitalaria y cuidados intensivos y mortalidad entre niños ingresados al servicio de urgencias pediátrico con diagnóstico de shock séptico refractario a volumen tratados inicialmente con dopamina o adrenalina.

## INTRODUCTION

Sepsis is a global health threat.^(1-7)^ Although the actual impact of sepsis is difficult to determine (due to a lack of adequate definitions and inadequate registration of the entity as a cause of death in global reports), it is considered to be the worldwide leading cause of death in childhood^(7)^ accounting for up to 60% of deaths in children under 5 years of age.^(2-6)^ In Argentina, few epidemiological data on sepsis in children are available; however, it has been estimated that sepsis represents one of the main causes of death in this population.^(8)^

Timely identification and treatment of sepsis may reduce mortality and minimize severe sequelae in survivors.^(1-6)^ Survival and length of hospital stay have improved through the adherence to guidelines such as those proposed by the American College of Critical Care Medicine (ACCM)^(9)^ and Pediatric Advance Life Support (PALS).^(10)^ Nevertheless, compliance with these standards by clinicians is relatively poor in high-income countries and in Latin America.^(10,11)^ One reason may be that many aspects of these guidelines remain controversial.^(9)^ An important issue is the choice of the first-line vasoactive agent (dopamine versus epinephrine) in children with fluid-refractory septic shock (FRSS). In spite of different randomized controlled pediatric clinical trials evaluating this issue,^(12,13)^ it remains a matter of debate.^(14,15)^ The current pediatric guidelines published in 2017 propose starting the administration of fluid boluses at a rate of 20mL/kg up to 60mL/kg in the first 15 - 20 minutes after sepsis is recognized; if shock does not reverse, it is recommended not to delay inotropic/vasopressor drugs infusion using a peripheral line if no central access is available.^(9)^ Regarding the choice of first-line inotropic drugs, guidelines suggest starting with epinephrine but dopamine is also an option.^(9)^

Considering that this choice is still an unaddressed issue and that the current trend is to prioritize epinephrine, it is necessary to characterize the clinical profile of children who are admitted to the emergency department with a diagnosis of FRSS and who are treated with currently considered first-line inotropic agents during the first hour of treatment.

The main aim of this study was to analyze the clinical outcome of children with FRSS treated with dopamine or epinephrine as a first-choice vasoactive agent at a pediatric emergency department. Specific objectives were to determine use of and time on invasive mechanical ventilation (IMV), length of hospital stay, length of intensive care unit (ICU) stay, time on vasoactive drug infusion, and mortality in both groups.

## METHODS

A retrospective cohort study was conducted at a national pediatric referral hospital. Our center receives more than 600,000 visits annually, of which approximately 95,000 are at the emergency department. The emergency department has 80 beds for observation, a specific area for resuscitation (“shock room”) with five beds equipped for critical patients, and immediate access to five pediatric ICUs with a total of 86 beds.

All patients consecutively admitted to the emergency department with a diagnosis of FRSS were included. Septic shock patients were defined as all children admitted to the shock room by an emergency physician with fever, tachycardia, and suspicion of infection associated with one or more of the following signs of tissue hypoperfusion: weak or bounding peripheral pulses; prolonged (> 2 - 3 seconds) or “flash” capillary refill; cold or warm extremities; low urinary output (< 1mL/kg/hour); and sensory disturbances (drowsiness, confusion, lethargy, etc.). FRSS was defined as all patients who, admitted with a diagnosis of septic shock, met one or more of the following criteria during initial treatment: infusion of 60mL/Kg of fluids or more; clinical signs of fluid overload (pulmonary rales and/or cough, hepatomegaly and/or third heart sound); poor general status, defined as the presence of one or more of the following signs/symptoms (arterial hypotension - systolic and/or diastolic blood pressure < than the 3rd percentile for age -, restlessness, sensory depression - Glasgow ≤ 13 -, and cyanotic/mottled appearance).

All patients were treated following the ACCM care guidelines^(9,16)^ in force during the study period and treatment was coordinated by an emergency department and/or ICU physician. Patients with a diagnosis of primary or secondary cardiomyopathy; those with a non-resuscitation order, patients receiving more than one inotropic/vasopressor drug from initiation; and/or those for whom a different drug was indicated (e.g., dobutamine or norepinephrine) were excluded. Data were collected from the medical records of all children admitted to the emergency room diagnosed with septic shock who required inotropic support after fluid administration between July 2009 and November 2017.

The data were collected in an ad hoc spreadsheet and then managed using RedCap (Research Electronic Data Capture) and Statistical Package for Social Science (SPSS), version 21. Both numerical and categorical variables are described using measures of central tendency (medians and their corresponding interquartile range due to the nonparametric distribution of the data obtained and frequency tables). For comparisons, the Mann-Whitney U and χ^2^ tests were used. To determine the level of statistical significance, an alpha error of 5% was accepted, which is equivalent to a p value less than 0.05. As this was a descriptive study, an estimation of the sample size was not performed. Because the main objective of observational studies is to generate hypotheses, the main results are reported according to recent recommendations on measures of effect and confidence intervals. The research protocol was approved by the Institutional Review Board.

## RESULTS

**Figure 1:**
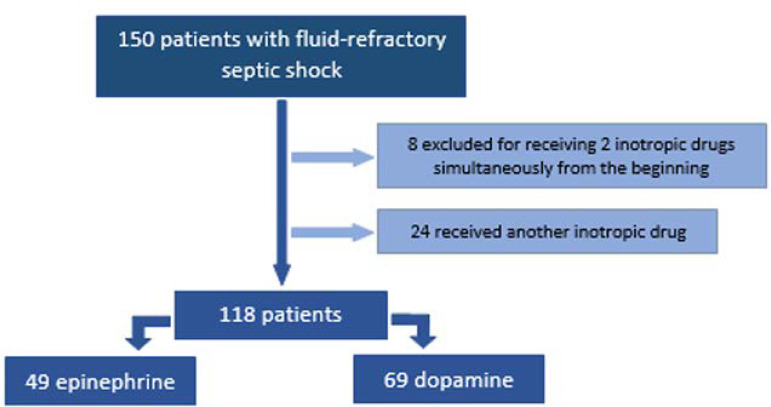
Patients admitted to the study.

Of the 150 patients admitted during the study period, 118 children met the inclusion criteria (Figure 1). The characteristics of the analyzed population are shown in table 1. The group that received dopamine more often had underlying disease (p = 0.032) or oncological disease (p = 0.007), treatment with immunosuppressants (p = 0.003), and positive blood cultures (p = 0.04) (Table 1). Regarding interventions for FRSS (Table 2), a significant difference was found in antibiotics administration during the first 60 minutes of treatment, with better adherence in the Dopamine Group (p < 0.001). No significant differences were found in time of inotropic drugs use, use of IMV, days on IMV, length of ICU stay, length of hospital stay, or mortality between groups (Table 3). Based on the differences in the percentage of patients with oncological disease between the groups, an additional comparison was made excluding these patients. As seen in table 4, when analyzing children without oncological disease (81 patients, 42 in the Epinephrine Group and 39 in the Dopamine Group), it was found that those receiving dopamine or epinephrine had a similar clinical outcome.

**Table 1:** Comparison of the demographic and clinical characteristics of children with fluid-refractory septic shock in both groups

Population variables	Epinephrine Group n = 49	Dopamine Group n = 69	p value*†
Male	31(63)	39 (57)	0.46*
Age (months)	63 (19 - 92)	81 (31 - 144)	0.09†
Chronic condition			
Yes	27 (55)	52 (75)	0.032*
Oncological	7 (14)	30 (43)	0.007*
With immunodeficiency (n = 117)	13 (27)	37 (54)	0.003*
With source of infection	39 (80)	54 (78)	0.8*
Source			
Digestive	18 (37)	16 (23)	
Respiratory	6 (12)	21 (30)	
Skin and soft parts	11 (22)	10 (15)	
Urinary	2 (4)	2 (3)	
Associated with catheter	1 (2)	4 (6)	
Central nervous system	1 (2)	1 (1)	
Arterial hypotension at baseline	20 (45)	21 (30)	0.2*
Positive blood cultures	11 (22)	27 (39)	0.04*
Germ in blood culture			
Gram-negative bacillus	6 (55)	12 (44)	
Methicillin-resistant *Staphylococcus aureus*	2 (18)	6 (22)	
Methicillin-sensitive *Staphylococcus aureus*	1 (9)	2 (7)	
Other	2 (18)	7 (27)	

* χ^2^ test; † Mann-Whitney U-test. Results expressed as n (%) or median (interquartile range).

**Table 2:** Comparison of interventions during the treatment of fluid-refractory septic shock

Treatment interventions	Epinephrine Group n = 49	Dopamine Group n = 69	p value*
Antibiotics in the first 60 minutes (n = 117)	36 (73)	68 (99)	< 0.001*
Reason for indication of the inotropic drug (n = 113)	47	66	
Fluid-refractory (60mL/kg)	38 (81)	34 (52)	
Signs of fluids overload	0	6 (9)	
Poor general condition	9 (19)	26 (39)	
Intubation in the emergency room (n = 116)	10 (20)	7 (10)	0.1*

* χ^2^ test. Results expressed as n (%) or n.

**Table 3:** Comparison between the variables of both groups

	Epinephrine Group n = 49	Dopamine Group n = 69	p value*†	OR (95%CI)
Death	5 (10)	9 (13)		1.32 (0.41 - 4.21)
IMV	19 (39)	28 (41)		1.07 (0.50 - 2.28)
IMV days	4	5,5	0.104†	
Days of hospitalization	11	13	0.554†	
Days of inotropic drugs	2	2	0.714†	
Days at ICU	4	4	0.748†	

OR - odds ratio; 95%CI - 95% confidence interval; IVM - invasive mechanical ventilation; ICU - intensive care unit.

* χ^2^ test; † Mann-Whitney U-test. Results expressed as n (%) or median in days.

**Table 4:** Comparison between the variables of both groups, excluding patients with oncological disease

	Epinephrine Group n = 42	Dopamine Group n = 39	p value*†	OR (95%CI)
Death	5 (12)	4 (10)		1.18 (0.29 - 4.76)
IMV	18 (43)	17 (44)		0.97 (0.40 - 2.33)
IMV days	3.5 (1 - 7)	6 (4.5 - 10.5)	0.062†	
Days of hospitalization	10 (6 - 15)	14	0.149†	
Days of inotropic drugs	1.5 (1 - 3)	2 (1 - 3)	0.239†	
Days at ICU	4 (2 - 6)	4 (2 - 8)	0.341†	

OR - odds ratio; 95% CI - 95% confidence interval; IVM- Invasive mechanical ventilation; ICU - intensive care unit.

* χ^2^ test; † Mann-Whitney U-test. Results expressed as n (%) or median in days.

## DISCUSSION

Our study shows that children with FRSS admitted to our emergency department who were treated either with dopamine or epinephrine as the first-line vasoactive drug had a similar clinical outcome considering relevant variables for patients who develop sepsis: need for and time on IMV, use of inotropic drugs, length of stay in the pediatric ICU, and mortality.

The strength of our study is that it is, to our knowledge, the first to describe a large population of children with septic shock analyzing outcome after inotropic therapy with two different regimens of inotropic support in a pediatric emergency department. In children, the evidence comparing both drugs is very limited, since most publications describe studies conducted in the ICU setting,^(12.13)^ where previously performed interventions cannot be clearly identified.

Another important aspect to consider is that our study was conducted at a national tertiary referral hospital with a large volume of patients, allowing us to obtain a heterogeneous group of patients that included a large number of children with chronic conditions, a population susceptible to developing sepsis in the course of their underlying disease. Likewise, the patients were treated by an experienced healthcare team trained in the management of these patients using current septic shock guidelines. The characteristics of the study population and the treating team should be considered when generalizing the results.

Among the limitations are the study design that did not reach the level of available randomized clinical trials. The retrospective observational design was chosen to meet our objectives because the pediatricians of our hospital had a change in practice during the recent period, in accordance with the changes in the recommendations for the management of sepsis in children worldwide that enable the use of epinephrine as a first-line drug.^(2,9,12)^ Considering the design of the study, it is likely that there has been a selection bias in terms of the drug used, related to the personal preferences of the treating physician and to the changes that occurred over time (as a result of the publication of the new guidelines). Dopamine has historically been recommended as the drug of choice in FRSS;^(9.16)^ therefore it was the drug used at our department until 2015. However, recent clinical trials on FRSS discussed in the introduction showed superiority of epinephrine over dopamine. Ventura et al. found a higher risk of mortality and secondary infections in children admitted to the ICU and treated with dopamine as the first choice for FRSS (20% of those treated with dopamine versus 7% of those who received epinephrine).^(12)^ They also found that the administration of epinephrine by a peripheral or intraosseous route was associated with better survival compared to dopamine.^(12)^ A similar trend was found by Ramaswamy et al., who reported that epinephrine was more effective than dopamine in resolving shock in the first hour of resuscitation, and that the epinephrine group had a lower Sequential Organ Function Assessment (SOFA) score on day 3 and after without organic dysfunction.^(13)^

In adults, a recent meta-analysis concluded that there is a benefit related to survival, the rate of adverse effects, and the hemodynamic profile with the use of epinephrine over dopamine in patients with septic shock.^(17)^ In adult patients, higher mortality and more frequent development of cardiac arrhythmias were associated with the use of dopamine for the treatment of septic shock. Other unwanted effects of dopamine are its immunosuppressive action and increased pulmonary shunt.^(18,19)^

The above evidence would explain the current trend to prefer epinephrine over dopamine among clinicians who care for children with FRSS.^(9)^ In any case, although the current trend is to prefer epinephrine as the first option, this debate has not been concluded. The results of current clinical trials have been criticized because of small sample sizes and especially the different dose regimens of drugs administered.^(14)^ In our series, we could not describe the doses of the different drugs, which is an added limitation to the interpretation of the results. Another important limitation of the study is that there were differences in demographic and clinical characteristics of the groups exposed to one or the other inotropic drug. This may be because during the study period, there were changes in the management of patients with oncological disease at our hospital. Since December 2015, the hospital has had a Comprehensive Oncology Patient Care Center (*Centro de Atención Integral del Paciente Oncológico*) that, between 8:00 am and 4:00 pm on weekdays is in charge of the emergencies in these children. This change coincided with the gradual incorporation of epinephrine as a first-line drug for the treatment of FRSS by clinicians, which is why children with oncological diseases were less prevalent in the Epinephrine Group. In the same sense, due to the duration of the study, several changes, such as staff training, the acquisition of more experience, and the implementation of care protocols and cards for the safe administration of drugs, could have affected the results.

When we excluded oncological patients in a subanalysis of the series, we found that clinical outcomes was similar in both groups, suggesting that “oncological disease” is not a factor causing major bias in the results. The difference observed in the indication of antibiotics between the two groups could be due to the change of the medical records from paper to electronic, causing the time of administration of the medication to be imprecise due to the lack of immediate availability of a computer. These aspects are additional limitations related to the study design.

It is difficult to evaluate adherence to new recommendations based on our results, although it is likely that, during the first years of the study, the treating team used dopamine as the drug of choice and that a proportion, due to personal preferences, did not change their actions despite the changes in published guidelines, which is why we found patients treated with dopamine in recent years.

No adverse effects of epinephrine (increased peripheral vascular resistance leading to decreased cardiac output and tissue perfusion, hypertension, tachycardia, necrosis due to capillary leakage, and, in preterm infants, hyperglycemia and increased lactate) and of dopamine (pulmonary vasoconstriction, tachycardia, arrhythmias, bradycardia, nausea, vomiting, hypothyroidism in preterm infants, and tissue necrosis),^(20)^ were found in the review of the medical records of the patients. In this retrospective study, this may have been due to reporting bias.

Despite these limitations, we believe that our results should be considered in future discussions on recommendations related to the indication and appropriate use of inotropic drugs in the treatment of sepsis in emergency departments within the first hour of its recognition. Although the current trend prioritizes the use of epinephrine, in the clinical context of this study, the impact of dopamine was similar when considering important clinical outcomes.

## CONCLUSIONS

Our study did not observe differences in the need for or time of invasive mechanical ventilation or inotropic drug use, length of intensive care unit stay and hospital stay, or mortality in children with fluid-refractory septic shock treated with epinephrine *versus* dopamine as the inotropic drug during initial resuscitation. At our center, both drugs appear to be useful for children with fluid-refractory septic shock.
